# TRANSLATING SCIENCE INTO PRACTICE: APPLYING IMPLEMENTATION SCIENCE TO STREAMLINE FUTURE HIH ROLL-OUT

**DOI:** 10.1093/geroni/igad104.0540

**Published:** 2023-12-21

**Authors:** Reza Yousefi Nooraie, Jennifer Sullivan, Lindsey Zimmerman, Marlena Shin, Emily Franzosa, Porooshat Dadgostar, Adele Levine, Derek Darcy

**Affiliations:** University of Rochester, Rochester, New York, United States; Center of Innovation in Long Term Services and Supports (LTSS COIN), Providence, Rhode Island, United States; National Center for Posttraumatic Stress Disorder, Menlo Park, California, United States; VA Boston Healthcare System, Boston, Massachusetts, United States; Icahn School of Medicine at Mount Sinai, New York City, New York, United States; University of Rochester Medical Center, Rochester, New York, United States; Boston University School of Public Health, Boston, Massachusetts, United States; Canandaigua VA Medical Center, Canandaigua, New York, United States

## Abstract

The delay between establishing evidence-based practice and translating effective practices is well documented. This presentation will focus on the application of implementation science principles to the Department of Veterans Affairs (VA) Hospital-In-Home program using a staged approach. The stages are: 1) assessing current program implementation, 2) measuring organizational readiness with new sites, 3) developing tools and resources to assist new HIH sites, and 4) working with new sites interested in adopting the HIH program using a participatory implementation planning process guided by system dynamics. In this session we will profile our use of theories, models and frameworks including the Consolidated Framework for Implementation Research (CFIR) and Reach Effectiveness Adoption Implementation Maintenance (REAIM) framework. The CFIR Expert Recommendation for Implementing Change (ERIC) Mapping Tool will be introduced as useful to identifying strategies to overcome barriers to implementation. We will apply the participatory approaches for implementation planning, including Implementation Mapping, development of Implementation Logic Models, and Group Model Building to plan for the implementation of HIH in prospective sites. Key implementation science concepts and techniques will be introduced, along with a description of how participatory approaches will facilitate informed planning and stakeholder engagement in future implementation, adaptation, and roll-out of an evidence-based systemic program. Key anticipated outputs include a framework for the core components of HIH intervention, a catalog of important determinants of HIH implementation success, a profile of existing strategies to enhance implementation and sustainability, and a roadmap for the participatory planning of HIH implementation in future contexts.

